# Rapid Purification of Endotoxin-Free RTX Toxins

**DOI:** 10.3390/toxins11060336

**Published:** 2019-06-12

**Authors:** Ondrej Stanek, Jiri Masin, Radim Osicka, David Jurnecka, Adriana Osickova, Peter Sebo

**Affiliations:** 1 Institute of Microbiology of the CAS, Videnska 1083, 142 20 Prague, Czech Republic; stanek@biomed.cas.cz (O.S.); david.jurnecka@biomed.cas.cz (D.J.); osickova@biomed.cas.cz (A.O.); sebo@biomed.cas.cz (P.S.); 2 Faculty of Science, Charles University, Hlavova 2030, 128 43 Prague, Czech Republic

**Keywords:** endotoxin, lipopolysaccharide, RTX toxins, Triton X-100, Triton X-114

## Abstract

Cytolytic leukotoxins of the repeat in toxin (RTX) family are large proteins excreted by gram-negative bacterial pathogens through the type 1 secretion system (T1SS). Due to low yields and poor stability in cultures of the original pathogens, it is useful to purify recombinant fatty-acylated RTX cytolysins from inclusion bodies produced in *E. coli*. Such preparations are, however, typically contaminated by high amounts of *E. coli* lipopolysaccharide (LPS or endotoxin). We report a simple procedure for purification of large amounts of biologically active and endotoxin-free RTX toxins. It is based on the common feature of RTX cytolysins that are T1SS-excreted as unfolded polypeptides and fold into a biologically active toxin only upon binding of calcium ions outside of the bacterial cell. Mimicking this process, the RTX proteins are solubilized from inclusion bodies with buffered 8 M urea, bound onto a suitable chromatographic medium under denaturing conditions and the contaminating LPS is removed through extensive on-column washes with buffers containing 6 to 8 M urea and 1% Triton X-100 or Triton X-114. Extensive on-column rinsing with 8 M urea buffer removes residual detergent and the eluted highly active RTX protein preparations then contain only trace amounts of LPS. The procedure is exemplified using four prototypic RTX cytolysins, the *Bordetella pertussis* CyaA and the hemolysins of *Escherichia coli* (HlyA), *Kingella kingae* (RtxA), and *Actinobacillus pleuropneumoniae* (ApxIA).

## 1. Introduction

Repeat in toxin (RTX) leukotoxins/cytolysins are a large family of pore-forming and immunomodulatory toxins of gram-negative pathogens belonging to the genera *Bordetella*, *Escherichia*, *Kingella*, *Moraxella*, *Morganella*, *Photorhabdus*, *Proteus*, *Vibrio*, and the *Pasteurellaceae* family. RTX toxins exert cytotoxic activities on a broad spectrum of eukaryotic host cells and share several characteristic features, such as: (i) the presence of a pore-forming hydrophobic domain; (ii) the requirement for activation by a posttranslational fatty-acyl modification accomplished by a cognate toxin activating acyltransferase (RtxC); (iii) the presence of an extensive C-terminal calcium-binding domain that consists of acidic glycine-rich repeats exhibiting a nonapeptide consensus sequence X-(L/I/F)-X-G-G-X-G-(N/D)-D; and (iv) the presence of an unprocessed C-terminal secretion signal involved in the export of the RTX protein out of the bacterial cell through a type I secretion system (T1SS) apparatus [1].

Among the best-characterized RTX toxins is the bi-functional adenylate cyclase toxin-hemolysin (CyaA) of *Bordetellae* that plays an important role in the airway colonization capacity and virulence of the whooping cough agent *Bordetella pertussis* [2,3]. The C-terminal hemolysin part of CyaA permeabilizes various host cells by cation-selective transmembrane pores, while the N-terminal adenylyl cyclase (AC) enzyme domain of CyaA translocates into cell cytosol and once activated by intracellular calmodulin, it subverts cellular signaling by catalyzing the unregulated conversion of cytosolic ATP to cAMP [4,5].

Another prototypic RTX cytolysin is α-hemolysin (HlyA), which is produced by numerous pathogenic as well as commensal *Escherichia coli* isolates. At higher toxin doses, the transmembrane pores formed by HlyA can provoke colloid-osmotic (oncotic) lysis of eukaryotic cells, while at lower concentrations the permeabilization of cells by HlyA interferes with host cell signaling pathways and can provoke apoptotic cell death [6]. Similarly, the emerging pathogen *Kingella kingae* secretes the membrane-damaging hemolysin RtxA involved in development of osteoarticular infections and infective endocarditis in children and adults [7]. *Actinobacillus pleuropneumoniae*, the etiological agent of swine pleuropneumonia, secretes the ApxIA hemolysin exhibiting strong hemolytic and cytotoxic activities [8,9].

Structural and functional studies of these RTX family cytolysins are often limited by their low yields from supernatants of cultures of the respective pathogens. An efficient method for obtaining of large quantities of the RTX toxins is their overexpression in recombinant *E. coli* cells together with their cognate RtxC toxin activating acyltransferases. This usually yields the formation of inclusion bodies from which the acylated RTX cytolysins are conveniently solubilized into 8 M urea buffers for further purification under denaturing conditions (6 to 8 M urea buffers). The biologically active cytolysins are then obtained by a more than ten-fold dilution of the urea-denatured toxin in a calcium-containing buffer. This mimics the natural process of calcium-triggered folding of the RTX cytolysin molecule upon exit of the translocating RTX polypeptide from the bacterial T1SS conduit into the calcium-rich (~2 mM Ca^2+^) host body fluids [10]. However, high amounts of contaminating *E. coli* outer membrane lipopolysaccharide (LPS) often limit the use of such-purified RTX cytolysin preparations in cellular signaling assays or toxin immunomodulatory activity studies. Indeed, LPS is recognized by the TLR4 receptor complex [11] that triggers signal transduction leading to NF-κB activation and pro-inflammatory cytokine release by monocytes and macrophages [12]. As a result, many insufficiently purified recombinant proteins were found to trigger TLR4 signaling until their cytokine-inducing activity was attributed to contamination by LPS [13,14,15,16,17]. Therefore, a number of processes has been developed to remove LPS from protein samples, including a two-phase extraction, chromatography on LPS affinity resins, ultrafiltration, hydrophobic interaction chromatography, ion exchange chromatography, and membrane adsorption [18]. The most universally applicable of these processes then appears to be the two-phase LPS extraction method with Triton X-114 [19,20,21,22].

Here we report a procedure specifically devised for simple and rapid purification of large amounts of LPS-free RTX toxins.

## 2. Results and Discussion

### 2.1. Purification of LPS-free RTX Toxins

**Figure 1:**
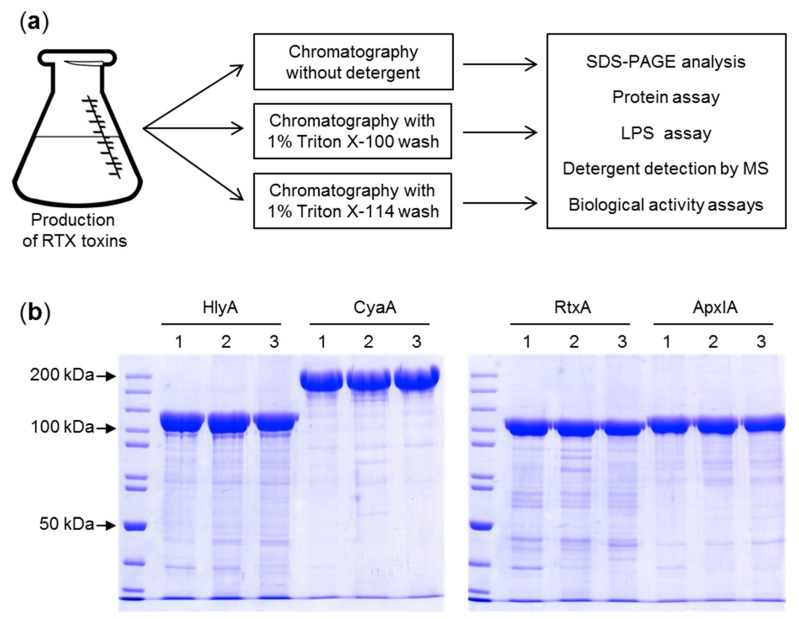
Purification of the repeat in toxin (RTX) toxins. (**a**) Schematic representation of the purification protocol. RTX toxin-producing *E. coli* cells were harvested, resuspended in buffer and divided in three equal aliquots that were processed in parallel, with, or without the indicated 1% detergent in the column wash buffer. (**b**) The indicated RTX toxin samples were separated by 7.5% SDS-PAGE and visualized by Coomassie blue staining. 1—purification without a detergent wash; 2—purification including a 1% Triton X-100 wash; 3—purification including a 1% Triton X-114 wash.

Previously, several methods for reduction of LPS contamination of purified CyaA toxin preparations were devised, including extensive washing of toxin-containing inclusion bodies with buffers containing 2 M urea or 1% (*v*/*v*) N-octyl-glucoside [23], removal of LPS from phenyl-sepharose-bound CyaA by 60% (*v*/*v*) isopropanol washes [24,25,26] or re-chromatography of purified CyaA on hydroxyapatite [27]. However, these purification procedures are laborious and typically reduce the yield or the specific activity of the purified toxin. Therefore, we aimed to develop a simplified single step purification procedure (Figure 1a) that would be generally applicable for removal of LPS from urea-solubilized RTX toxin preparations. We took advantage of the unique capacity of T1SS-secreted RTX proteins to undergo calcium driven folding into a biologically active conformation upon simple dilution of the denaturant. Therefore, the entire purification process was performed under denaturing conditions in buffers containing 8 M urea and thus could be performed at room temperature without the risk of protein degradation. Use of 8 M urea buffers also provided the opportunity to exploit the strong binding of the highly negatively charged denatured CyaA (pI 4) to the positively charged groups of DEAE-Sepharose at pH 8 and the high affinity interaction of the denatured His-tagged recombinant HlyA, RtxA, and ApxIA proteins with Ni-NTA agarose beads, respectively. Following protein extract loading on the chromatographic matrices, removal of trapped LPS was achieved by washing of the toxin-loaded resins beds with 10 volumes of 1% (*v*/*v*) solution of the non-ionic detergents Triton X-100 or 1% (*v*/*v*) Triton X-114 in 8 M urea. The detergent was next removed by resin washes with 5 bed volumes of urea buffer without detergent, before CyaA was eluted in 8 M urea buffer pH 8 containing 200 mM NaCl and the HlyA, RtxA, or ApxIA proteins were eluted with 250 mM imidazole in 8 M urea buffer, respectively (Figure 1b). We also performed reference purifications, in which the resins beds were washed with 10 bed volumes of urea buffers without Triton detergents.

As shown in Table 1, when the RTX proteins were purified without including the 1% Triton X-100 or Triton X-114 detergents into the washing 8 M urea buffers, the amounts of LPS detected in the eluted toxin samples ranged from 4 × 10^5^ to 1 × 10^6^ endotoxin units (EU) per mg of the RTX protein, irrespective of whether a DEAE-Sepharose or Ni-NTA agarose column matrix was used. Inclusion of 1% Triton X-100 into column wash buffer reduced the LPS amounts in the purified RTX protein samples by three to four orders of magnitude down to ≤115 EU/mg. Inclusion of 1% Triton X-114 in the wash buffer reduced the LPS content in the purified samples of the RTX proteins even more to ≤25 EU/mg (Table 1). Hence, use of either detergent in the buffer used to wash either of the two used purification matrices allowed a significant reduction of the LPS content in the final preparations of all four RTX toxins.

**Figure 2:**
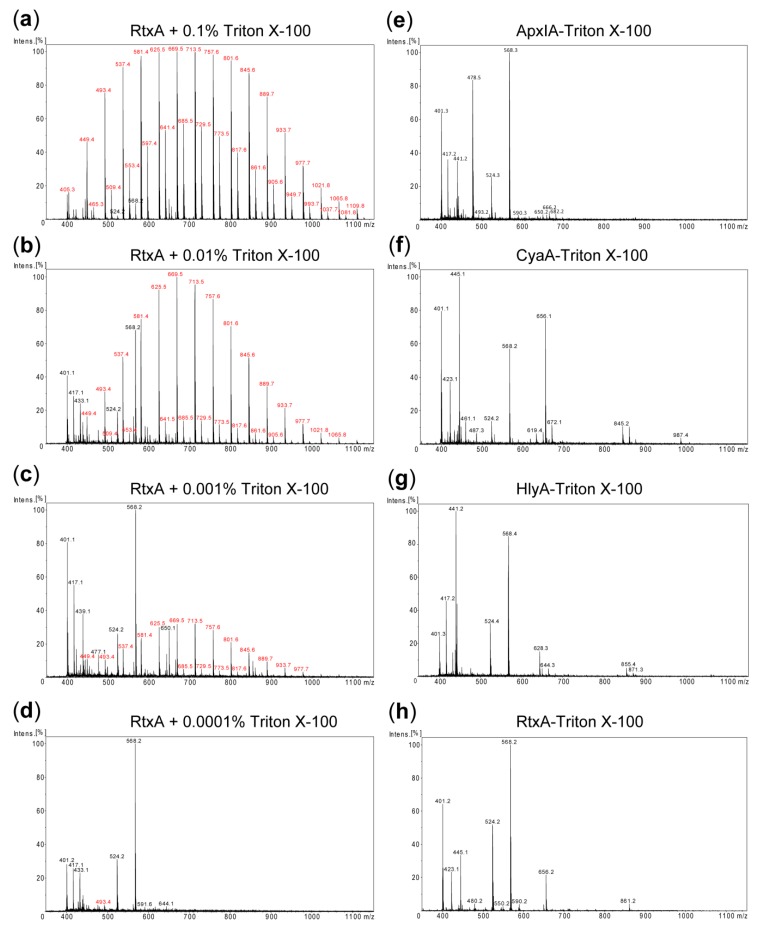
Detection of residual detergent in purified RTX toxin samples. (**a**–**d**) RtxA (1 mg/mL) purified without the detergent was spiked with Triton X-100 at concentrations decreasing from 0.1 to 0.0001% and analyzed by matrix-assisted laser desorption ionization-time-of-flight (MALDI-TOF). (**e**–**h**) MALDI-TOF spectra of the RTX toxin samples purified using the 1% Triton X-100 column wash. The m/z values of ions corresponding to Triton X-100 components are printed in red. The remaining ions represent adducts of the matrix and other small molecular mass contaminants. In each panel an ion with maximum intensity was taken as 100%.

Since traces of the used Triton detergents might potentially interfere with biological activities of the purified RTX toxins and confound cell cytotoxicity assays, it was important to examine whether the detergent was efficiently removed upon excessive column washing with detergent-free 8 M urea buffer. Towards this aim matrix-assisted laser desorption ionization-time-of-flight (MALDI-TOF) mass spectrometry (MS) was employed to detect any residual contamination of the purified RTX toxins by trace amounts of Triton X-100. First, the detergent-free RtxA protein sample (1 mg/mL) was spiked with Triton X-100 in the range of from 0.1 to 0.0001% and analyzed by MALDI-TOF MS. As shown in Figure 2a–d, Triton X-100-specific peaks were unambiguously detected in the MALDI-TOF spectra of RtxA samples containing 0.001% or higher Triton X-100 concentrations. In contrast, no such peaks were present in the spectra of the RTX proteins purified without the use of detergent (Figure S1), or in the spectra obtained for the RTX proteins that were purified using Triton X-100 in the column wash solution (Figure 2e–h). Similar MS results were obtained when Triton X-114 was used to remove endotoxin from the RTX toxin preparations (Figure S2). Hence, if traces of the detergent were still present in the ~10 µM protein preparations, the concentrations of remaining Triton detergent were below the detection limit of the method (~0.001%, or ~16–20 µM). Given that purified toxin stocks are further diluted over 100-fold when assayed for biological activity, protein or LPS content, such low amounts of detergent are well below the effective cytolytic concentration [28] and are very unlikely to interfere in any of these assays.

### 2.2. The LPS-Free RTX Toxins Are Cytotoxic on Different Cell Types

**Figure 3:**
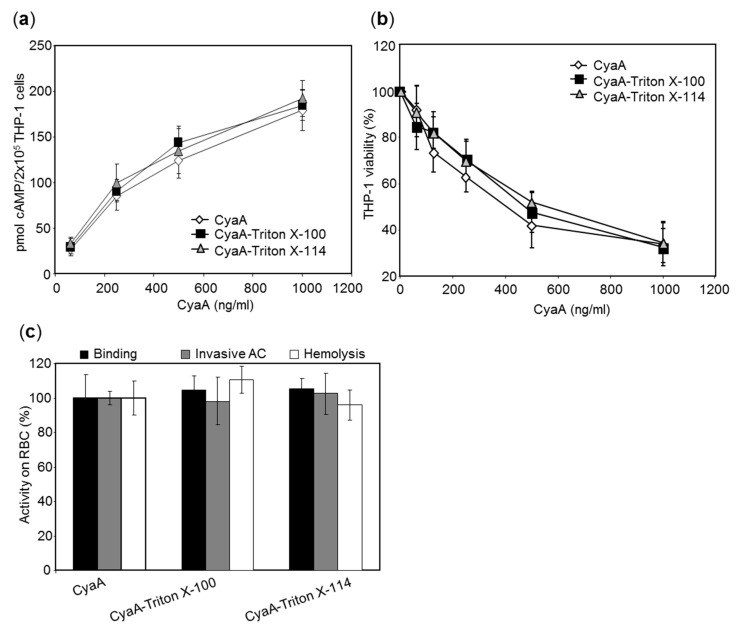
Toxin activities of CyaA purified in the presence or absence of detergent. (**a**) CyaA toxin (62.5–1000 ng/mL) purified with or without a 1% Triton X-100 wash of the chromatographic resin was incubated with 2 × 10^5^ THP-1 cells in 100 µL of D-MEM medium and cAMP intoxication was assessed by determining the intracellular concentration of cAMP generated in cells after 30 min of incubation with CyaA at the indicated concentrations. Average values ± standard deviations from three independent experiments performed in duplicates are shown. (**b**) Cells were incubated with the CyaA toxins at 37 °C for 2 h and the number of viable cells was determined using the CCK-8 assay. The viability of mock-treated cells (buffer only) was taken as 100%. The percentages of viable cells represent average values ± standard deviations from two independent experiments performed in triplicates. (**c**) Sheep erythrocytes were incubated at 37 °C with 1 μg/mL of the CyaA toxins and after 30 min, aliquots were taken for determination of the cell-associated adenylyl cyclase (AC) activity and of the AC activity internalized into erythrocytes and protected against digestion by externally added trypsin. For determination of hemolytic activity, sheep erythrocytes (5 × 10^8^/mL) were incubated at 37 °C in the presence of 10 μg/mL of the CyaA toxins and erythrocyte lysis was measured after 3 h as the amount of released hemoglobin by photometric determination at 541 nm (A_541_). Each activity is expressed as percentage relative to the activity of CyaA purified in the absence of Triton and represents average value ± standard deviation from at least two independent determinations performed in duplicates with two different toxin preparations.

We investigated whether the RTX toxins purified in the presence of the Triton detergents preserved their biological activities. The CyaA preparations purified in the presence or absence of Triton were analyzed using human THP-1 monocytes as model cells expressing the integrin CD11b/CD18 receptor [29,30]. Sheep erythrocytes then served as model cells lacking CD11b/CD18. As summarized in Figure 3a, at equal protein concentrations the CyaA toxins purified in the presence of Triton X-100 or Triton X-114 intoxicated THP-1 monocytes by cAMP with the same capacity as the CyaA toxin purified in the absence of the detergents. Similarly, all three CyaA preparations exhibited a comparable capacity to reduce THP-1 viability, determined as the loss of mitochondrial dehydrogenase activity (Figure 3b). Finally, CyaA purified in the presence of detergents bound the surface of erythrocytes, translocated the AC domain to their cytosol, and formed hemolytic pores in the cell membrane with the same efficacy as the CyaA toxin purified in the absence of the detergents (Figure 3c). All these results demonstrate that the detergent-involving procedure for purification of LPS-free CyaA yielded a fully biologically active toxin.

**Figure 4:**
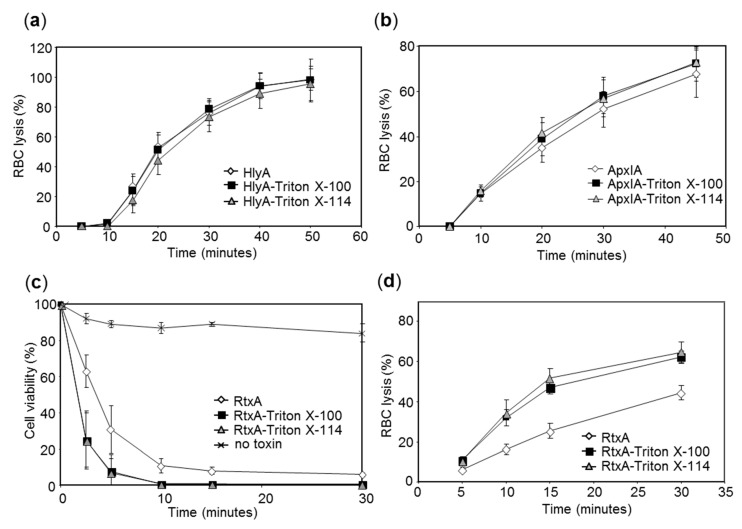
Cytolytic activities of purified HlyA, ApxIA, and RtxA toxins. (**a**,**b**) Washed sheep erythrocytes (5 × 10^8^/mL) were incubated at 37 °C with 20 ng/mL of HlyA (**a**) or 1 µg/mL of ApxIA (**b**) and erythrocyte lysis was measured in time as the amount of released hemoglobin by photometric determination at 541 nm (A_541_). Average values ± standard deviations from four independent determinations are shown. (**c**) HLaC-78 cells (1 × 10^6^/mL) were incubated with 1 µg/mL of RtxA for indicated time at 37 °C. Cell viability was determined by a vital dye staining using 1 µg/mL of Hoechst 33258 followed by flow cytometry. The initial viability of cells incubated without RtxA was taken as 100%. Each point represents the mean value ± standard deviation of three independent experiments. (**d**) Sheep erythrocytes (5 × 10^8^/mL) were incubated at 37 °C in the presence of 200 ng/mL of RtxA and erythrocyte lysis was measured as above.

Next, we examined pore-forming (hemolytic) activity of the HlyA and ApxIA hemolysins purified in the presence or absence of Triton X-100 or of Triton X-114. As shown in Figure 4a,b, the hemolytic potency of both RTX hemolysins was not affected at all by the use of Triton X-100 or Triton X-114 detergents in column wash during toxin purification and removal of LPS from toxin preparations had no effect on their cytolytic capacities.

Intriguingly, when the cytotoxic activity of purified RtxA was tested using the laryngeal HLaC-78 squamous cells, which are exquisitely susceptible to RtxA-mediated killing [31], the cytotoxic activity of the LPS-depleted RtxA-Triton X-100 and RtxA-Triton X-114 proteins (1 µg/mL) was importantly higher than the toxicity of the RtxA protein purified without the use of detergent in column washes (Figure 4c). A similar result was observed when the hemolytic capacity of purified RtxA toxin was assessed on sheep erythrocytes, where the LPS-depleted RtxA-Triton X-100 and RtxA-Triton-X114 proteins were more potent as hemolysins. Given the low residual Triton X-100 concentration (<16 µM) in the purified RtxA-Triton X-100 preparation (~10 µM) it appears unlikely that the traces of the detergent present at <16 nM concentration upon the 1000-fold dilution of the toxin stock solution into cellular suspensions could have had any stabilizing effect on the final ~10 nM RtxA protein and potentiate its activity. Rather, the presence of micromolar concentrations of contaminating *E. coli* LPS (~100–200 µg/mL or ~10–20 µM) in the RtxA sample purified without the use of detergent in column wash might have reduced toxin activity. Indeed, it was previously observed that RtxA has to be diluted from 8 M urea solutions into the assay buffer immediately before adding to cells and preincubation of the renatured toxin in the assay buffer causes a rapid loss of cytotoxic activity of RtxA [31]. It is tempting to speculate that in the absence of chaotropic concentrations of urea the hydrophobic sites of RtxA may bind the lipid A moiety of LPS and this may affect the capacity of RtxA to interact with the target cells.

## 3. Conclusions

In conclusion, using four different recombinant RTX toxins as example, we present here a simple, rapid, and universal method of purification of endotoxin-depleted recombinant RTX toxins from 8 M urea extracts of *E. coli* inclusion bodies. The purification is performed at room temperature under denaturing conditions and is easily scalable. The included LPS depletion step consists in extensive on-column wash of the protein with 1% solution of Triton X-100 or Triton X-114 detergents. The detergent is effectively removed by extensive resin rinsing with 8 M urea buffer prior to RTX toxin elution, which yields concentrated RTX toxin preparations of superior homogeneity and high specific biological activity. In the case of RtxA toxin of *K. kingae* the results indicate that on-column removal of contaminating LPS by detergent wash may enhance the specific cytotoxic activity of RtxA. The described procedure opens the way to large production of endotoxin-free RTX protein preparations needed for structural and functional studies of these intriguing bacterial toxins.

## 4. Materials and Methods

### 4.1. Expression and Isolation of RTX Toxins

Plasmid pT7CACT1 was used for co-expression of the *cyaC* and *cyaA* genes for production of recombinant CyaC-activated CyaA [32]. To produce the RtxC-activated RtxA toxin equipped with a C-terminal double 6xHis purification tag, the plasmid pT7rtxC-rtxA was used [31]. For production of HlyC-activated HlyA with a C-terminal double 6xHis tag, the *rtxC* and *rtxA* genes of pT7rtxC-rtxA were replaced by the *hlyC* and *hlyA* genes to yield the pT7hlyC-hlyA expression vector. For production of the acylated ApxIA toxin having 6xHis tags on both the N-terminal and C-terminal ends, the pET28bapxIC-apxIA construct was used [9].

The toxins were produced in *E. coli* BL21/pMM100 (HlyA, CyaA, and RtxA) or *E. coli* Rosetta 2 (ApxIA) cells in 1000 mL cultures in MDO medium (yeast extract, 20 g/L; glycerol, 20 g/L; KH_2_PO_4_, 1 g/L; K_2_HPO_4_, 3 g/L; NH_4_Cl, 2 g/L; Na_2_SO_4_, 0.5 g/L; and thiamine hydrochloride, 0.01 g/L) at 37 °C following IPTG induction (0.5 mM) for 3 h. The pellets of harvested bacteria were resuspended in 30 mL of 50 mM Tris-HCl pH 8.0, sonicated and split into three aliquots of 10 mL. Inclusion bodies from each aliquot were washed in 10 mL of 50 mM Tris-HCl (pH 8.0), 2 M urea buffer and then solubilized at room temperature for 30 min in 8 M urea in 5 mL of 50 mM Tris-HCl pH 8.0 containing either (i) 1% (*v*/*v*) Triton X-100 (Sigma-Aldrich, St. Louis, MO, USA), (ii) 1% (*v*/*v*) Triton X-114 (Sigma-Aldrich, St. Louis, MO, USA) or (iii) no detergent. The urea extracts were then cleared by centrifugation at 25,000× *g* for 30 min at 4 °C.

### 4.2. Purification of CyaA

The cleared urea extracts of CyaA were supplemented with NaCl to a final concentration of 50 mM and loaded at room temperature onto columns with 10 mL of packed DEAE-Sepharose resin pre-equilibrated with 8 M urea, 50 mM Tris-HCl pH 8.0, 120 mM NaCl buffer that contained no detergent, or was supplemented with 1% (*v*/*v*) of Triton X-100 or 1% (*v*/*v*) of Triton X-114, respectively. Contaminating *E. coli* components were removed by washing of the columns with 10 bed volumes of the respective equilibration buffer. Detergent was next removed by 5 bed volume washes with 8 M urea, 50 mM Tris-HCl pH 8, and 120 mM NaCl. Finally, CyaA was eluted with 8 M urea, 50 mM Tris-HCl pH 8, and 200 mM NaCl, and the fractions were analyzed by SDS-PAGE (Figure 1b).

### 4.3. Purification of HlyA, ApxIA, and RtxA

The His tag-equipped RTX hemolysins HlyA, ApxIA, and RtxA were purified by immobilized metal ion affinity chromatography on Ni-NTA agarose (GE Healthcare BioSciences, Pittsburgh, PA, USA). The cleared urea extracts of inclusion bodies were supplemented with NaCl to 300 mM final concentration and loaded at room temperature on 5 mL Ni-NTA agarose columns equilibrated with 8 M urea, 50 mM Tris-HCl pH 8.0, 300 mM NaCl buffer without detergent, or with the same buffer containing 1% (*v*/*v*) of Triton X-100 or Triton X-114, respectively. Contaminating *E. coli* components were removed by extensive washing of the column with 10 bed volumes of the given equilibration buffers. Detergent was removed by washes with 5 bed volumes of 8 M urea, 50 mM Tris-HCl pH 8.0, 300 mM NaCl, and 30 mM imidazole, and the RTX toxins were eluted with 250 mM imidazole in the same buffer. EDTA was added to 1 mM and the pooled fractions were extensively dialyzed against 100 volumes of 8 M urea, 50 mM Tris-HCl, and 300 mM NaCl to remove imidazole and the homogeneity of the proteins was analyzed by SDS-PAGE (Figure 1b).

### 4.4. Determination of LPS Levels and Protein Concentrations

The LPS levels in protein samples were determined by the Chromogenic LAL assay (Charles River Endosave, Charleston, SC, USA) and protein concentration was determined by the Bradford assay using BSA as the calibration standard.

### 4.5. Detection of Residual Detergent

MALDI-TOF MS of protein preparations was performed using an Autoflex III (Bruker Daltonics, Bremen, Germany) instrument with mass spectra acquisition in the positive reflection mode within a scan range of 300–4000 Da. Prior to MS analysis the RTX toxin samples (50 µg) were desalted by protein MacroTrap (Optimize Technologies, Oregon City, OR, USA) and eluted with 100 μL of 80% ACN, 0.1% TFA. Desalted proteins were dried via vacuum centrifugation and dissolved in 30 μL of 5% ACN, 0.1% TFA (*v*/*v*). The samples were then mixed on the target with α-Cyano-4-hydroxycinnamic acid and dried at room temperature. Results were analyzed using Data Analysis 4.1 (Bruker Daltonics).

### 4.6. Determination of Hemolytic Activity on Sheep Erythrocytes

Sheep erythrocytes (LabMediaServis, Jaromer, Czech Republic) were washed, resuspended to 5 × 10^8^/mL in 50 mM Tris-Cl, 150 mM NaCl, and 2 mM CaCl_2_, pH 7.4 (buffer A) and incubated for 3 h with 10 µg/mL of CyaA, before the amount of released hemoglobin was measured as absorbance at 541 nm [33]. The lytic activity of CyaA purified without detergent was taken as 100%. The hemolytic activities of HlyA, RtxA, and ApxIA were measured as hemoglobin release over time of incubation of 5 × 10^8^/mL of washed sheep erythrocytes in buffer A with 20 ng/mL of HlyA, 200 ng/mL of RtxA, or 1 µg/mL ApxIA, respectively. As a negative control, the hemolytic activity of toxins was measured in buffer containing 5 mM EDTA instead of 2 mM CaCl_2_ and no lysis was observed (not shown).

### 4.7. Cell Binding and Invasive Activities of CyaA on Sheep Erythrocytes

The AC enzymatic activities of CyaA were determined in the presence of 1 µM calmodulin as previously described [34]. One unit of AC activity corresponds to 1 µmol of cAMP formed per min at 30 °C, pH 8.0. Cell invasive AC enzyme activity was measured as previously described [35], by determining the AC enzyme activity protected against externally added trypsin upon internalization into cytosol of sheep erythrocytes. Erythrocyte binding of CyaA was determined as previously described [35] by determining the amount of cell-associated (membrane bound) AC enzyme activity. Activity of CyaA purified without detergent was taken as 100%.

### 4.8. Human Cell Lines

Human monocytes THP-1 (ATCC, number TIB-202) and laryngeal HLaC-78 squamous cells [36] were cultured at 37 °C in a humidified air/CO_2_ (19:1) atmosphere in RPMI 1640 (Sigma-Aldrich, St. Louis, MO, USA) supplemented with 10% (*v*/*v*) fetal calf serum (FCS) (GIBCO Invitrogen, Grand Island, NY, USA) and antibiotic antimycotic solution (0.1 mg/mL streptomycin, 100 U/mL penicillin, and 0.25 mg/mL amphotericin; Sigma-Aldrich, St. Louis, MO, USA).

### 4.9. cAMP Elevation and Viability Assays on THP-1 Cells

Prior to assays, the RPMI 1640 medium used for THP-1 cultivation was replaced by D-MEM medium (1.9 mM Ca^2+^) without FCS and the cells were allowed to rest in D-MEM for 1 h at 37 °C in a humidified 5% CO_2_ atmosphere. For intracellular cAMP assay, 2 × 10^5^ THP-1 cells were incubated at 37 °C with CyaA for 30 min in D-MEM, the reaction was stopped by addition of 0.2% (*v*/*v*) Tween-20 in 100 mM HCl, the samples were boiled for 15 min at 100 °C, neutralized by addition of 150 mM unbuffered imidazole and cAMP was measured by a competitive immunoassay as previously described [37]. For cell viability assay, the THP-1 (2 × 10^5^) cells were incubated with indicated concentration of CyaA at 37 °C for 2 h in a humidified air/CO_2_ atmosphere and the number of viable cells was determined as WST-8 reduction using the CCK-8 assay (Dojindo Laboratories Kamimashiki-gun, Japan). The viability of mock-treated control cells was taken as 100%.

### 4.10. Determination of RtxA Cytotoxicity on HLaC-78 Cells

Viability of HLaC-78 cells upon incubation with RtxA was determined as previously described [31]. Briefly, HLaC-78 cells were harvested, washed in HEPES-buffered salt solution (10 mM HEPES (pH 7.4), 140 mM NaCl, 5 mM KCl) supplemented with 2 mM CaCl_2_, 2 mM MgCl_2_ and diluted with the same buffer to 1 × 10^6^ cells/mL. The cells were then incubated at 37 °C with 1 µg/mL of purified RtxA samples for the indicated times. Cell viability was determined by a vital dye staining with 1 µg/mL of Hoechst 33258 and flow cytometry on a FACS LSR II instrument (BD Biosciences, San Jose, CA, USA). Data were analyzed using the FlowJo software (Tree Star, Ashland, OR, USA) and appropriate gatings were used to exclude cell aggregates and dying/dead cells (Hoechst 33258-positive staining).

## Figures and Tables

**Table 1:** RTX toxin concentrations, lipopolysaccharide (LPS) contaminations and total amounts of the proteins obtained from one liter of culture.

RTX Toxin ^1^	Without Detergent Wash			1% Triton X-100 Wash			1% Triton X-114 Wash		
	Conc. ^2^ (mg/mL)	LPS ^3^ (EU/mg)	Total ^4^ (mg)	Conc. ^2^ (mg/mL)	LPS ^3^ (EU/mg)	Total ^4^ (mg)	Conc. ^2^ (mg/mL)	LPS ^3^ (EU/mg)	Total ^4^ (mg)
CyaA	1.3	>4 × 10^5^	6	1.0	12	6	1.2	8	6
RtxA	2.0	>1 × 10^6^	8	2.1	115	8	2.0	25	8
HlyA	1.4	>1 × 10^6^	4	1.3	95	4	1.4	15	4
ApxIA	1.9	>1 × 10^6^	8	2.1	85	8	1.6	15	8

^1^ adenylate cyclase toxin-hemolysin (CyaA), RtxA, α-hemolysin (HlyA) and ApxIA toxins were purified as described in detail in Materials and Methods. ^2^ Protein concentration was determined by the Bradford assay. ^3^ LPS content was determined by the Chromogenic LAL assay. ^4^ Total protein amount.

## Supplementary Materials

The following are available online at https://www.mdpi.com/2072-6651/11/6/336/s1, Figure S1: MALDI-TOF spectra of the RTX toxin samples purified without the use of a detergent wash, Figure S2: Detection of residual detergent in purified RTX toxin samples.
