# Supplementary material for: Rapid Purification of Endotoxin-Free RTX Toxins
Source: Toxins (Basel). 2019 Jun 12;11(6):336. doi: 10.3390/toxins11060336 (PMC6628407; doi:10.3390/toxins11060336)
Supplement: Supplementary file 1 [file toxins-11-00336-s001.pdf]

Supporting Information: Rapid Purification of Endotoxin-Free RTX Toxins

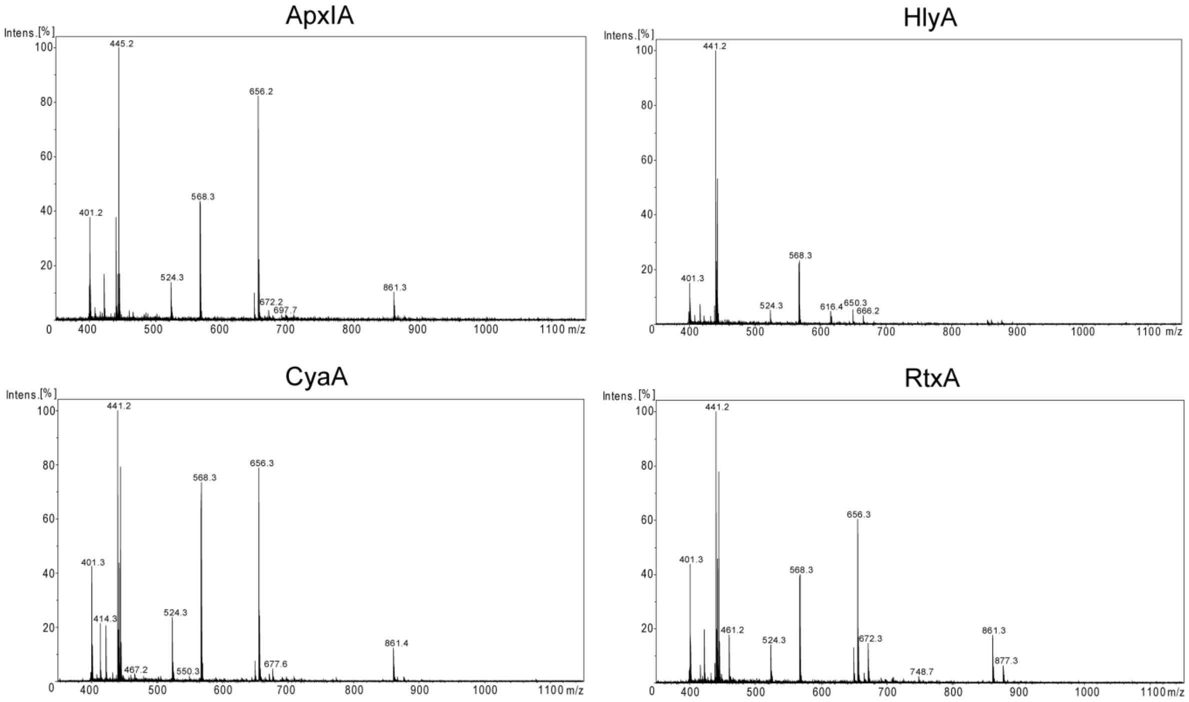

Figure S1. MALDI-TOF spectra of the RTX toxin samples purified without the use of a detergent wash. The detected ions represent adducts of the matrix and other small molecular mass contaminants. No ions corresponding to Triton X-100 were detected. In each panel an ion with maximum intensity was taken as 100%.

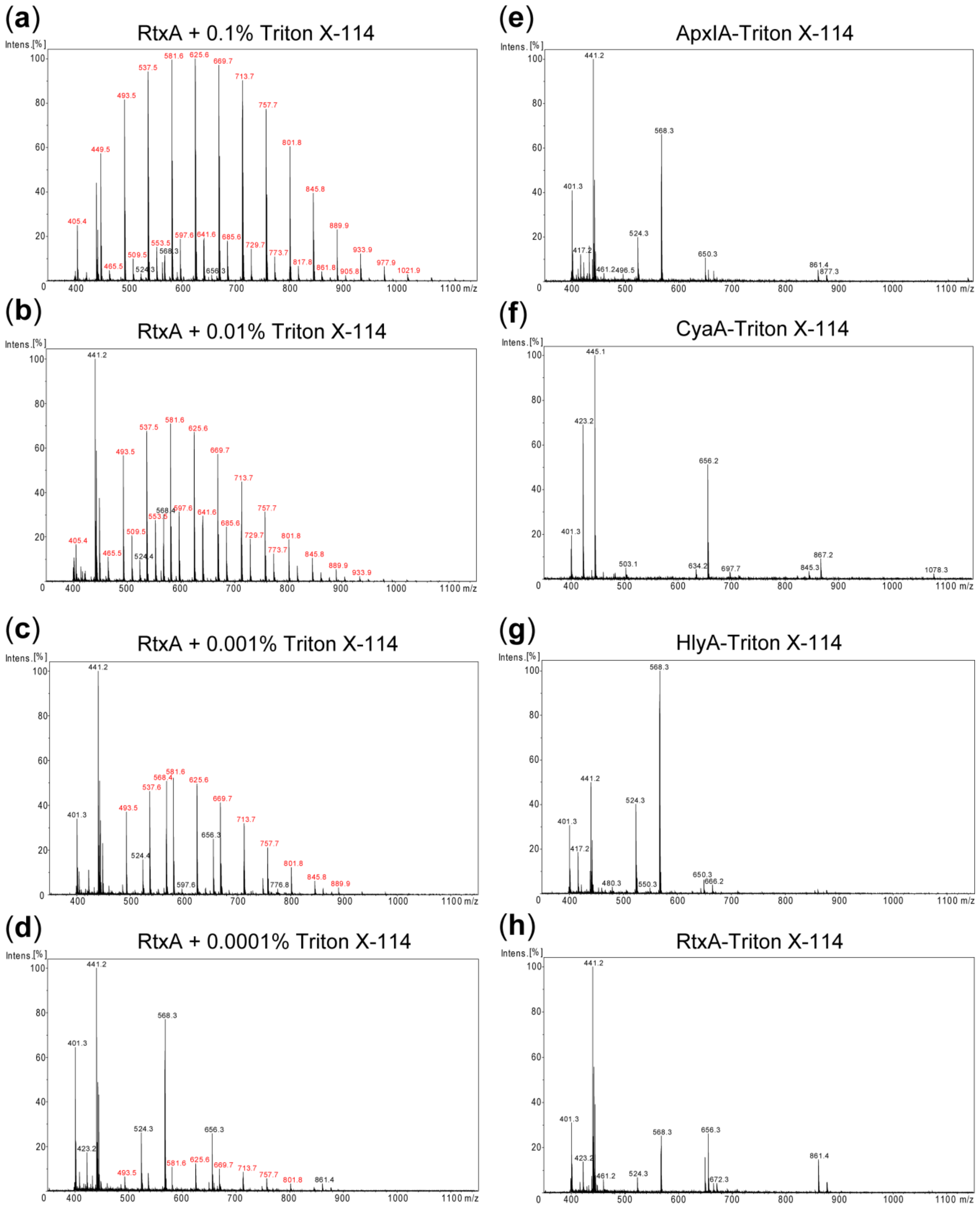

Figure S2. Detection of residual detergent in purified RTX toxin samples. (**a**–**d**) RtxA (1 mg/mL) purified without the detergent was spiked with Triton X-114 at concentrations decreasing from 0.1 to 0.0001% and analyzed by MALDI-TOF. (**e**–**h**) MALDI-TOF spectra of the RTX samples purified using the 1% Triton X-114 column wash. The *m*/*z* values of ions corresponding to Triton X-114 components are printed in red. The remaining ions represent adducts of the matrix and other small molecular mass contaminants. In each panel an ion with maximum intensity was taken as 100%.
